# Casting histone variants during mammalian reproduction

**DOI:** 10.1007/s00412-023-00803-9

**Published:** 2023-06-22

**Authors:** Germaine Karam, Antoine Molaro

**Affiliations:** Genetics, Reproduction and Development Institute (iGReD), CNRS UMR 6293, INSERM U1103, Université Clermont Auvergne, Clermont-Ferrand, France

**Keywords:** Histone, Variants, Reproduction, Mammals, Evolution, Parental-effect

## Abstract

During mammalian reproduction, germ cell chromatin packaging is key to prepare parental genomes for fertilization and to initiate embryonic development. While chromatin modifications such as DNA methylation and histone post-translational modifications are well known to carry regulatory information, histone variants have received less attention in this context. Histone variants alter the stability, structure and function of nucleosomes and, as such, contribute to chromatin organization in germ cells. Here, we review histone variants expression dynamics during the production of male and female germ cells, and what is currently known about their parent-of-origin effects during reproduction. Finally, we discuss the apparent conundrum behind these important functions and their recent evolutionary diversification.

## Introduction: casting histone variants for the next blockbuster

In eukaryotes, genetic information is organized into chromatin. The basic unit of chromatin is the nucleosome composed of histone proteins wrapping ~ 147 bp of DNA. Most nucleosomes contain four types of replication dependent histone proteins H2A, H2B, H3, and H4 (thereafter referred to as core histones) (Phillips and Johns 1965; Kornberg 1974). They assemble as an octamer: one H3-H4 tetramer interacting with two H2A-H2B dimers (Arents et al. 1991; Luger 1997). An additional, structurally distinct, histone protein called H1 binds to the linker DNA located between nucleosomes and further contributes to genome packaging into “chromatosomes” (Simpson 1978; Thoma et al. 1979). Core histones are some of the slowest evolving proteins in eukaryotes, being nearly identical between distantly related species (Malik and Henikoff 2003; Molaro and Drinnenberg 2018; Talbert and Henikoff 2021). This is thought to be the result of strong purifying natural selection maintaining their essential functions (Rooney et al. 2002; Piontkivska et al. 2002; Eirín-López et al. 2004). Indeed, histones regulate access to genetic information by impeding protein interactions with DNA and by altering chromatin states through post-translational modifications (Kornberg and Lorch 2020; Millán-Zambrano et al. 2022).

Nevertheless, eukaryote histones did not remain evolutionary inert since their birth. In fact, stand-alone histone variants repeatedly arose from their core counterparts either by de novo gene duplication or via sub-functionalization of existing histone paralogs (Malik and Henikoff 2003; Talbert et al. 2012; Draizen et al. 2016; Osakabe and Molaro 2023). All extant histones have described variants differing by only a few amino-acids (a.a.), as seen for the pan-eukaryote H3 variant H3.3, to having gained entire additional domains as is the case for macroH2A in metazoans (reviewed in Malik and Henikoff 2003; Talbert and Henikoff 2021).

Unlike core histones, histone variants can be deposited into nucleosomes independently of DNA replication and influence genome regulation in post-mitotic cells (reviewed in Marzluff et al. 2008; Martire and Banaszynski 2020; Talbert and Henikoff 2021). Consequently, over the last 2 billion years, eukaryotic histone variants took on essential functions during DNA repair, transcription or chromatin remodeling and play major roles during both normal and disease development (reviewed in Maze et al. 2014; Zink and Hake 2016; Martire and Banaszynski 2020; Talbert and Henikoff 2021). For example, genetic ablation of many histone variants can have severe developmental consequences in both mice and human (e.g., H2A.Z: Faast et al. 2001; H3.3: Jang et al. 2015). In addition, a growing number of studies have linked somatic histone variant mutations, or ectopic induction, to cancer progression (Wu et al. 2012; Schwartzentruber et al. 2012; Kallappagoudar et al. 2015; Buschbeck and Hake 2017; Nacev et al. 2019; Gomes et al. 2019; Chew et al. 2021).

Beside these canonical roles in the soma, histone variant functions have most diversified in the germline. While we focus here on mammals, this observation holds true across eukaryotes (Orsi et al. 2009; Jiang et al. 2020a; Osakabe and Molaro 2023). Germline cells express histone variant families with unique sequence features or repurpose ubiquitous variants towards functions not seen in somatic cells (Kimmins and Sassone-Corsi 2005; Santenard and Torres-Padilla 2009; Hoghoughi et al. 2018). This is seen for H2A.X which carries out its DNA repair function in the context of meiotic cross-overs and colocalizes, together with macroH2A, to the sex-body during male meiosis (Hoyer-Fender et al. 2000; Turner et al. 2001; Celeste et al. 2002; Fernandez-Capetillo et al. 2003; Pasque et al. 2012). This diversification likely stems from the highly specialized functions of chromatin landscapes during reproduction. First, chromatin states established during gametogenesis are essential for proper genome packaging and contribute to the inheritance of parent-of-origin information to the zygote (Kimmins and Sassone-Corsi 2005; van der Heijden et al. 2006; Teperek et al. 2016; Hanna and Kelsey 2017). Then, following fertilization, chromatin remodeling can template developmental transitions relying on genome accessibility (Santos and Dean 2004; Saitou et al. 2012; Bošković et al. 2014). Finally, the specification of future germ cells requires coordinated chromatin reprogramming events safeguarding future generations (Surani et al. 2004; Hackett et al. 2012; Matsui and Mochizuki 2014; Kurimoto and Saitou 2019).

Here, we review histone variants with unique functions in the mammalian germline with a focus on their function setting up parental chromatin landscapes with putative effects on embryonic development. We then discuss their evolutionary trajectories highlighting conserved *vs.* species-specific functions.

## Histone variants during male gametogenesis: stunt doubles steal the spotlight

Like their female counterparts, male germ cells arise from a population primordial germ cells (or PGCs) that colonize the gonads during embryonic development (Ginsburg et al. 1990). Major chromatin remodeling events occur during PGC development, including whole genome DNA methylation and histone modification reprogramming (Surani et al. 2004; Hackett et al. 2012; Matsui and Mochizuki 2014; Kurimoto and Saitou 2019). Probably owing to the technical difficulties of studying such a discrete cell population, we know surprisingly little about histone variants’ function in PGCs. For example, there have been no reports of PGC-specific mammalian histone variants and those investigated appear to carry out their somatic functions (Surani et al. 2004; Matsui and Mochizuki 2014; Kurimoto and Saitou 2019). For example, H2A.Z enrichment in PGC nuclei correlates with active transcription as seen in somatic cells (Hajkova et al. 2008) .

Nevertheless, once PGCs settle to establish spermatogonial stem cells (SSCs) and the process of spermatogenesis begins, histone variants take the center stage. This is due to the major chromatin remodeling events occurring both during meiosis (Turner 2015; Wang et al. 2017) and in post-meiotic germ cells (reviewed in Rathke et al. 2014). The highlight of this play during spermiogenesis is the replacement of histone-based nucleosomes with transition proteins and then protamines (Oliva and Dixon 1991). Yet, histone-to-protamine replacement is incomplete and a few nucleosomes are retained in mature sperm over a few genes and repeats (Erkek et al. 2013; Hammoud et al. 2009; Carone et al. 2014; Samans et al. 2014; Yamaguchi et al. 2018). Retained nucleosomes contain histone variants and can be post-translationally modified (Brunner et al. 2014; Luense et al. 2016). While there is much species-specific variation, these nucleosomes have the potential to contribute to chromatin-based paternal effects post-fertilization.

Considering the vast amount of histone variants identified in males, we separated them into their respective families. We also mostly cover those with specific expression in germ cells and/or carrying parental effects. Other recent reviews cover the function of ubiquitously expressed variants (Martire and Banaszynski 2020; Talbert and Henikoff 2021).

### H3 variants

**Fig. 1:**
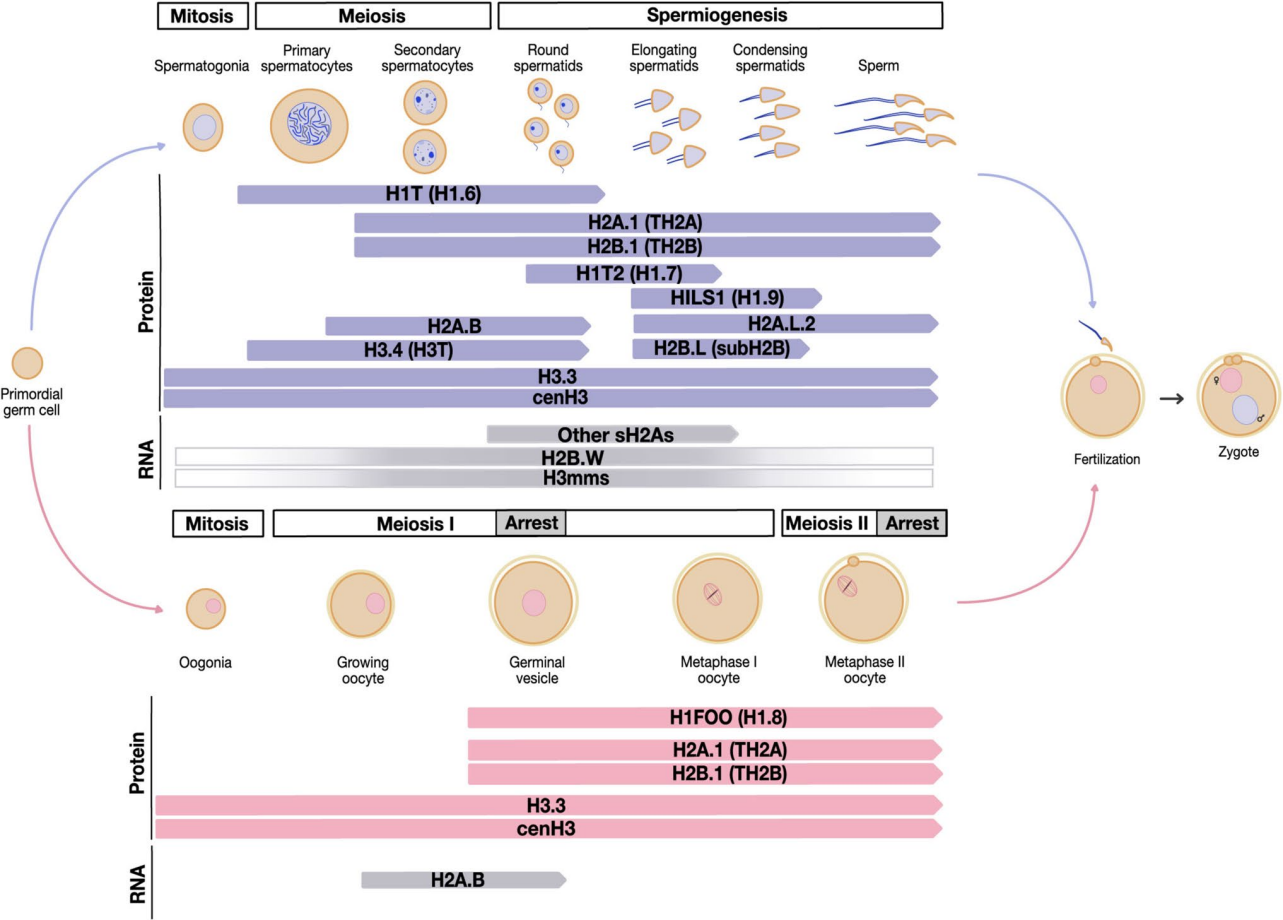
**Expression timing of mouse histone variants during gametogenesis.** The window of expression (RNA) or chromatin association (protein) is shown for histone variants discussed in this review. Stages of male (blue) or female (pink) germ cell differentiation are indicated above. H3mms and H2B.W expression has not been staged during gametogenesis. H3.3 and cenH3 are also included although their expression is not restricted to germ cells.

**H3.3** is one of the main H3 variants present during spermatogenesis, and it is found in retained nucleosomes in mature sperm (Fig. 1) (Bramlage et al. 1997; Hammoud et al. 2009; Erkek et al. 2013). In this context, it has been mapped to CpG-rich unmethylated promoters and shown to carry post-translational modifications, such as trimethylation of Lysine 4, 9, 27, and 36 in mice humans or bulls (Samans et al. 2014; Brunner et al. 2014; Luense et al. 2016; Jung et al. 2017). H3.3 is encoded by two genes — *h3fb3a* and *h3fb3b* — coding for identical proteins that differ from core H3s by only a few amino acids, most notably in their chaperone interaction domain (Talbert and Henikoff 2021). While complete *H3.3* KO are embryonic lethal, single KOs are viable and have non-overlapping expression and function during spermatogenesis (Couldrey et al. 1999; Yuen et al. 2014; Tang et al. 2015). H3.3A loss leads to mild sperm abnormality and sub-fertility suggesting that H3.3B alone is sufficient to complete spermatogenesis (Couldrey et al. 1999). On the other hand, *H3.3B* KO males are infertile and arrest after meiosis is completed (Fontaine et al. 2022). Although the precise molecular mechanisms behind this arrest are not fully understood, a recent study suggested that H3.3B might play a role in the chromatin remodeling of sex chromosomes, repeats, and piRNA clusters induced after meiosis (Fontaine et al. 2022). Yet, how these germ cell specific functions are tied to H3.3 post-translational modifications in mature sperm remains largely unknown.

**H3mm** variants were identified through phylogenetic analyses suggesting a recent origin in mice via repeated amplifications of H3.3 coding genes (Maehara et al. 2015). The mouse genome encodes 14 H3mms, and four of these — H3mm7, 8, 13, and 15 — are expressed in the testis, albeit not exclusively (Maehara et al. 2015). Mouse KO for *H3mm7* have altered muscle differentiation but defects during spermatogenesis were not reported (Harada et al. 2018). H3mms close sequence similarity to H3.3 suggest they might share similar deposition pathways with related functions during spermatogenesis. However, it remains to be shown if H3mms also contribute to mature sperm nucleosomes.

**H3.5** arose in the last common ancestor of great-apes (Hominoids) likely via retroposition of H3.3B (Schenk et al. 2011). Compared to H3.3, H3.5 differ by 5 a.a. and one of these sites, Leucine 103, is responsible for weakened hydrophobic interactions with H4 which decreases the stability of H3.5 nucleosomes (Urahama et al. 2016). In human, H3.5 is highly expressed in spermatogonia and is lost during meiosis I suggesting it might accumulate during cell division (Urahama et al. 2016; Shiraishi et al. 2018; Ding et al. 2021). So far, one study investigated H3.5 genomic localization in human testes, and found preferential accumulation at the 5′ end of genes. This indicates that H3.5 loading might occur over transcribed genes or contributes to chromatin opening during spermatogonial differentiation (Urahama et al. 2016).

**H3.X** and **H3.Y** seem also restricted to primates and are detected in human testes, but not exclusively (Wiedemann et al. 2010). They share most similarities with H3.3, from which they differ by 26 a.a. for H3.Y and 35 a.a. for H3.X; these concentrate at sites interacting with nucelosomal DNA (Wiedemann et al. 2010; Kujirai et al. 2017). Although their localization in the germline has never been investigated, H3.X/Y deposition in somatic cells relies on the chaperone HIRA and is associated with active transcription (Kujirai et al. 2016; Zink et al. 2017; Resnick et al. 2019). Interestingly, they are regulated by the transcription factor DUX4 that plays an important role in testis and zygote transcriptional regulation (Young et al. 2013; Resnick et al. 2019; Vuoristo et al. 2022). This suggests that H3.X/Y might play key roles in the germline developmental program that remains to be discovered.

**H3T (or H3.4).** In addition to H3.3 variants, mammalian genomes also encode a testis-specific H3, known as H3T (or H3.4). This variant shares more similarity with core H3 (e.g., H3.1) than H3.3 (Witt et al. 1996; Ueda et al. 2017), and its deposition is limited to cells entering meiosis (spermatocytes). Mice KO for *H3t* are infertile, and structural analyses suggested that H3t-containing nucleosomes favor chromatin loosening required for meiotic progression (Ueda et al. 2017). As discussed above, this function is also suggested for human H3.5, but unlike this variant, H3T is found in most mammals (see below). Thus, it would be interesting to understand whether H3T and H3.3 variants interact to regulate chromatin conformation during meiosis. **cenH3** defines chromosome centromeres in most eukaryotes (McKinley and Cheeseman 2016; Mellone and Fachinetti 2021). Although it is ubiquitously expressed, it performs specialized functions in the germline. Indeed, in male post-meiotic cells, cenH3 is retained at centromeres and carries-out paternal chromosome centromere inheritance through fertilization (reviewed by Das et al. 2017). As such, it is one of the few histone variants with clearly identified parental-effect molecular function. Interestingly, this function in sperm could help organize peri-centromeric chromatin into chromocenters which influence species-specific paternal genome remodeling following fertilization (Zalensky et al. 1993; Probst et al. 2007, 2009; van de Werken et al. 2014; Burton et al. 2020).

### H2A variants

**TH2A (or H2A.1)** is considered to be the major histone variant replacing core H2A in post-replicative germ cells (Trostle-Weige et al. 1982) (Fig. 1). In this context, TH2A shares some of the interacting partners and post-translational modifications of core H2A (e.g., H2AK119Ub Chen et al. 1998; Baarends et al. 1999; Hasegawa et al. 2015). However, TH2A is also uniquely phosphorylated at Thr127 concomitant to its deposition around centromeres during male and female meiosis (Shinagawa et al. 2014; Hada et al. 2017; Talbert and Henikoff 2021). *Th2a* KO have no reported phenotypes, but TH2A loss combined with its partner TH2B (H2B.1) causes improper meiotic exit and histone-to-protamine replacement (Shinagawa et al. 2015). While maternal contribution of TH2A and TH2B to the embryo is required for both paternal genome reprogramming and zygotic gene activation, whether they also contribute to paternal chromatin-based inheritance remains currently unknown (Shinagawa et al. 2014, 2015).

**Short H2As** probably win the award for most unusual variants in the male germline. These histones arose in placental mammals and are subject to unprecedented levels of evolutionary diversification (Molaro et al. 2018). They exist in four flavors — H2A.B, H2A.L, H2A.P, and H2A.Q — and are expressed sequentially during spermatogenesis. In mice, this begins with H2A.B during meiosis, then H2A.P and finally H2A.Ls in differentiating spermatids, H2A.Q being lost in mice (Govin et al. 2007; Ferguson et al. 2009; Soboleva et al. 2012; Molaro et al. 2018) (Fig. 1). Compared to core H2A, all four short H2As have highly divergent tails, histone fold domains, and are truncated at their C-terminal docking domain. These alterations greatly reduce the DNA wrapping, stability, and interactions of short H2A containing nucleosomes (Bao et al. 2004; Doyen et al. 2006; Syed et al. 2009; Arimura et al. 2013; Molaro et al. 2018; Kohestani and Wereszczynski 2021). How these structural features affect chromatin remodeling likely depends on the developmental context, e.g., induced in somatic cells *vs.* endogenous expression in germ cells (also reviewed in Jiang et al. 2020b).

**H2A.B** accumulates in meiotic spermatocytes but disappears from chromatin when haploid spermatids begin their differentiation. In mice, H2A.B appears to associate with actively transcribed genes where it has been proposed to contribute to RNA processing, most notably via its interaction with RNA and the splicing machinery (Soboleva et al. 2012, 2017). While *H2a.b* KO males display mild transcriptional and spermatogenesis defects, they show decreased nucleosome wrapping in post-meiotic cells, are sub-fertile, and sire litters with increased mortality (Anuar et al. 2019; Molaro et al. 2020). Embryonic development is altered upon paternal and maternal loss of H2A.B indicating a parental-effect function during mouse reproduction (Molaro et al. 2020).

**H2A.L.2** is broadly deposited in differentiating mouse spermatids concomitant with histone-to-protamine replacement (Govin et al. 2007; Barral et al. 2017). In this context, H2A.L.2 directly interacts with transition proteins helping unwrap DNA during protamine deposition. Despite the presence of 19 additional coding *H2a.l* paralogs in the mouse genome, H2A.L.2 function is essential and *H2a.l.2* KO mice are infertile (Barral et al. 2017).

Aside for these two examples, the function of short H2As remains mostly unexplored. Further molecular studies will be required to understand their partners, modifications, and whether they interact with one another during germ cell development.

Special mentions: **H2A.X**, **macroH2A**, and **H2A.Z**. These three H2As are some of the most studied variants owing to their essential functions during development and recent reviews thoroughly cover these histones (Herchenröther et al. 2023; Oberdoerffer and Miller 2023). Yet, beside their canonical roles, it is worth mentioning that all three histones are known to regulate the packaging and transcription of sex chromosomes during male meiosis (Mahadevaiah et al. 2001; Fernandez-Capetillo et al. 2003; Greaves et al. 2006). Akin to its somatic function, H2A.X is also directly involved in the signaling and repair of double strand breaks induced during meiosis I (Lichten 2001). While the molecular details of these meiosis-specific functions are complex and beyond the scope of this overview, they are well documented examples of histone variants functional repurposing in the unique context of germ cell development.

### H2B variants

**TH2B** **(or H2B.1**) is the main H2B variant present in both male and female germ cells and is likely present in nucleosomes retained in mature sperm (Mahadevaiah et al. 2001; Fernandez-Capetillo et al. 2003; Brock et al. 1980; Patankar et al. 2021; Singh and Parte 2021). TH2B shares ~ 85% identity with core H2B, with differences concentrated over the tail region thereby weakening DNA interactions (Pentakota et al. 2014). As mentioned above, when paired with *Th2a*, *Th2b* mouse KO display severe fertility defects. Yet, single *Th2b* KO showed that this variant played a key role in destabilizing nucleosomes during histone-to-protamine transition (Montellier et al. 2013). This function could be dependent on TH2B specific interaction with germ cell chromatin remodelers, as a tagged version of the histone display severe dominant negative sterility (Montellier et al. 2013). Which remodeling factor or nucleosome modifying enzyme might carry this function remains unknown.

**H2B.L** (**or subH2B**) is an unusual H2B variants that was first identified as part of the acrosomal region of bull sperm (Aul and Oko 2001). H2B.L is 4 amino acids shorter than core H2B and is subject to rapid evolutionary diversification in mammals (Aul and Oko 2001; Raman et al. 2022). Which function during reproduction might be driving this accelerated evolution is still unknown.

**H2B.W** is also unusual as it is the only H2B variant with an extended N-terminal tail (Churikov et al. 2004; Raman et al. 2022). It is most abundant in the testis and binds to telomeric chromatin when expressed in somatic cells; however, this function has not been tested in vivo (Churikov et al. 2004; Boulard et al. 2006). In most mammals, H2B.W is encoded by multiple paralogs — including a paralog previously known as H2B.M — and are the most rapidly diverging *H2B* genes identified to date (Raman et al. 2022).

### H1 variants

In mammals, there are six somatic variants (H1.1 to 5 and H1.10) and four that are exclusively found in the germline (H1.6 to 9) (Fan et al. 2003, 2005; Eirín-López et al. 2004; Ponte et al. 2017; Talbert and Henikoff 2021). While they have received less attention overall, these variants contribute nonetheless to the functional diversity of germ cell chromatin.

**H1T (or H1.6)** was first identified during rat spermatogenesis where its abundance exceeds 50% of all H1s (Bucci et al. 1982). In mice, H1T expression is restricted to meiotic and post-meiotic cells (Drabent et al. 1996, 1998). Recent studies have mapped H1T to transposable elements coated with DNA methylation, H3K9me3 and H4K20me3 suggesting it might be associated with their transcriptional repression (Mahadevan et al. 2020). Surprisingly, *H1t* KO male mice display no abnormalities during spermatogenesis (Drabent et al. 1996). Thus, it would be interesting to investigate how other testis-restricted H1s might functionally replace H1T (such as H1.9, see below), as observed for somatic H1s (Fan et al. 2003, 2005), and if species-specific features drive H1T functions.

**H1T2 (or H1.7)** is expressed following meiosis during spermatogenesis in round and elongating spermatids (Shalini et al. 2021). Albeit with some divergence, H1.7 and H1.9 share an extended C-terminal tail not found in other H1s (Tanaka et al. 2005). Unlike H1T, male mice with homozygous deletion of *H1.7* are infertile with sub-optimal histone to protamine replacement (Martianov et al. 2005; Tanaka et al. 2005). So far, H1.7 has not been found in mature sperm chromatin (Tanaka et al. 2005).

**HILS1 (or H1.9)** is the final testis-specific H1 variant expressed in elongating spermatids (Yan et al. 2003). In addition to being highly divergent compared to other H1s, H1.9 appears to be rapidly evolving in mammals (Su et al. 2013). A recent study showed that it induces relaxed chromatin states and is enriched over LINE-1 elements in spermatids (Mishra et al. 2018). How this function relates to species-specific histone-to-protamine exchange remains currently unknown (Mishra et al. 2018). However, since H1s are detected in mature sperm (Luense et al. 2016), which of these variants makes the final cut remains to be found.

## Female germ cell specific histone variants: maternal breakthrough roles

Unlike continuous meiosis in males, female gametes are produced through one wave of meiosis from PGCs and arrest twice during this process: first, at the end of meiotic prophase I at the germinal vesicle, or GV, stage (Mehlmann 2005); second, meiosis resumes at each ovulation cycle after puberty but stops at metaphase of meiosis II to produce fertilization competent oocytes (Mehlmann 2005).

These meiotic arrests are coupled to broad transcriptional quiescence and the establishment of non-canonical patterns of histone modifications (Moore et al. 1974; Kageyama et al. 2007; Dahl et al. 2016). In mice, H3K4me3 becomes distributed over broad domains at non-transcribed genic and intergenic regions through the action of MLL2 and contributes to transcriptional silencing (Dahl et al. 2016; Zhang et al. 2016; Hanna et al. 2018). H3K27me3 and H2AK119Ub also expand to intergenic regions (Zheng et al. 2016; Chen et al. 2021; Mei et al. 2021). Together, these post translational modification patterns contribute to a novel form of maternal non-canonical imprint regulating embryonic growth (Inoue et al. 2017; Hanna and Kelsey 2017; Mei et al. 2021). Yet, these non-canonical imprints seem to diverge between species. Most notably, human oocytes are devoid of such noncanonical patterns of H3K27me3 and H3K4me3 (Lu et al. 2021).

Both core and variant histones have been detected in the oocyte, either at the RNA or protein level (Wassarman and Mrozak 1981; Aoki et al. 1997; Torres-Padilla et al. 2006; Nashun et al. 2010; Shinagawa et al. 2014, 2015; Kong et al. 2018; Raman et al. 2022). Maternal histones will contribute to the repackaging of parental genomes shortly after fertilization towards zygotic genome activation (reviewed in Yang et al. 2015). Thus, histone function in female gametes can rarely be decoupled from their maternal-effect on the zygote. Core H2A, H2A.Z and macroH2A have all been detected in oocyte chromatin (Nashun et al. 2010; Liu et al. 2022). These histones delocalize from the maternal chromatin following fertilization and only reassociate with embryonic chromatin after zygotic genome activation (Nashun et al. 2010; Liu et al. 2022). Only H2A.X is loaded onto parental genomes around fertilization, and this activity depends on its unique C-terminal tail involved in DNA damage sensing (Nashun et al. 2010).

Likely due to the difficulties of studying such a discrete cell type, most female specific histone variants have not been functionally characterized besides their expression patterns. This includes the only H4 variant described in mammals, H4.G. It is detected in human ovaries and various tumors (Wassarman and Mrozak 1981; Long et al. 2019). Another example are the recently identified H2B variants H2B.K and H2B.N (Raman et al. 2022). In humans, their expression peaks during meiosis I and again following fertilization (Raman et al. 2022). Finally, the short H2A variant H2A.B is also found during female meiosis but its chromatin function in females remains completely unknown (Molaro et al. 2020). While these variants have yet to reveal their female breakthrough roles, we specifically discuss established maternal-effect variants in the following sections (Fig. 1).

**H3.3** is deposited in growing oocytes and becomes the main H3 variant up to their maturation (Torres-Padilla et al. 2006; Akiyama et al. 2011). As such it is the major carrier of post-translational modifications unique to the mouse oocyte (Akiyama et al. 2011). In this context, H3.3 accumulation gradually shifts from its canonical euchromatic pattern in growing oocytes to an enrichment at heterochromatic regions in the mature oocytes (Ishiuchi et al. 2021). H3.3 knockdown in mature oocytes leads to suboptimal development of early zygotes suggesting that maternal H3.3 deposition plays a critical role post-fertilization (Kong et al. 2018). This function is tied to paternal genome remodeling and activation (van der Heijden et al. 2005; Torres-Padilla et al. 2006; Santenard and Torres-Padilla 2009; Akiyama et al. 2011; Kong et al. 2018). Thus, H3.3 temporal function during gametogenesis and early embryonic development finely orchestrates parental chromatin remodeling around fertilization.

**CenH3** is also found in the chromatin of mature oocytes. There, it performs its unique germline function of centromere identification and inheritance as discussed in the previous section (Das et al. 2017). However, in females, cenH3 binding to centromeres has been proposed to contribute to the suppression of selfish chromosomes drive during asymmetric meiosis (Henikoff et al. 2001; Kursel and Malik 2018). This crucial function has profound consequences on cenH3 evolutionary trajectory discussed in the next section.

**TH2A (H2A.1)** and **TH2B (H2B.1)** variants are highly expressed and favored over their core counterparts in the oocyte. They are maternally deposited in the mouse zygote, and briefly induced upon genome activation. Maternal TH2A/TH2B contribute to the activation of the paternal genome after fertilization, possibly by inducing a more open chromatin structure compared to the core histones (Tanaka et al. 2001; Shinagawa et al. 2014).

**H1FOO (or H1.8)** is specifically induced at the GV stage where it almost entirely replaces core H1 up to the mature MII oocyte stage in mice (Tanaka et al. 2001; Gao et al. 2004). Upon fertilization, some H1FOOs are loaded onto the paternal genome (Tanaka et al. 2001). Given that H1FOO has a greater chromatosome mobility than H1, it could contribute to chromatin remodeling leading-up to zygotic genome activation (Teranishi et al. 2004; Hayakawa et al. 2012). However, considering that mature oocytes are transcriptionally inert, this remodeling function must be coupled to specific chromatin features of the zygote.

## Evolutionary trajectories of mammalian germline histone variants: spin-offs and sequels

**Fig. 2:**
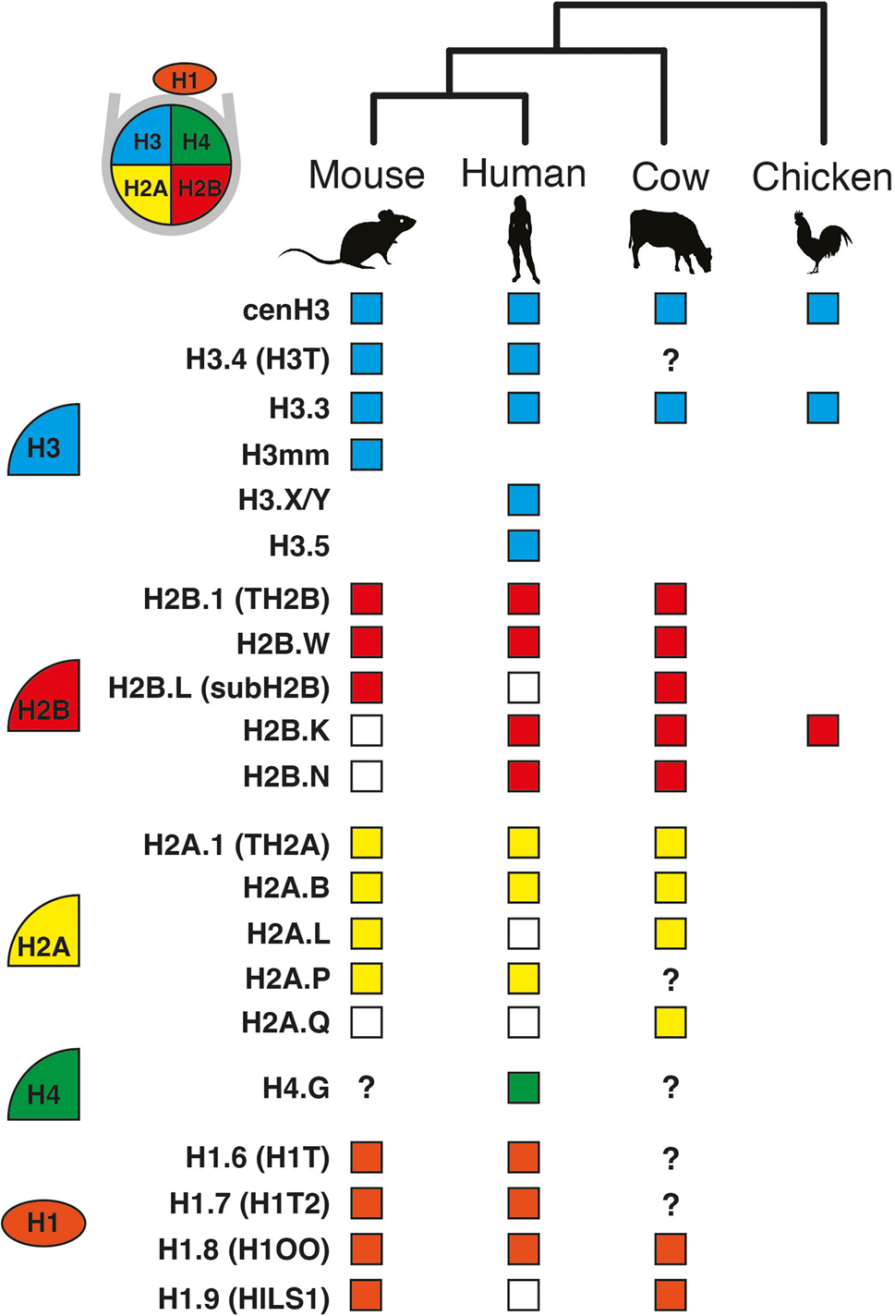
**Germline histone variants orthologs identified in mammals.** Histone variants with germline specific functions or expression are classified by type (H3, H2A, H2B, H4 and H1). Filled boxes indicate identified orthologous genes in the mouse, human, and cow genomes, with chicken used an outgroup. Empty boxes indicate pseudogenization or other secondary loss event. “?” denotes unresolved orthology. In the following paragraphs, we briefly discuss these evolutionary scenarios for germline histone variants.

From this overview, it is clear that when germline histone variants are investigated in details, all are found to be crucial for reproductive fitness. While this might be expected for variants sharing long evolutionary histories with our genomes, it is perhaps more surprising for recently evolved ones. Indeed, it is generally assumed that essential processes involve universally conserved players under strong purifying selection. However, reproduction is also a place of intense evolutionary tensions driving functional diversification (Moore and Haig 1991; Partridge and Hurst 1998; Martin and Hosken 2003; Crespi and Semeniuk 2004) (Fig. 2).

To begin, there are those ubiquitously expressed histone variants showing unique functions in germ cells (e.g., H2A.X, macroH2A). All are deeply conserved and have maintained steady evolutionary trajectories in mammalian genomes (Malik and Henikoff 2003; Talbert et al. 2012; Molaro and Drinnenberg 2018; Talbert and Henikoff 2021). One might anticipate their function in gametes or zygote genome packaging to also be conserved between mammals. The only exception is cenH3 which, despite its ancient birth in eukaryotes, displays accelerated rates of evolution between closely related species. Part of this diversification might be the direct consequence of cenH3's role in chromosome drive suppression during female meiosis (Chmátal et al. 2014; also reviewed in Lampson and Black 2017; Kursel and Malik 2018). In this context, cenH3 centromere function is hypothesized to re-establish fair chromosome segregation when “cheating chromosomes” take advantage of female asymmetrical meiosis for inclusion in the oocyte. As such, cenH3 evolutionary trajectory is directly linked to its germline function. Nevertheless, future research will probably uncover novel species-specific layers of chromatin regulation involving other ancient variants in the germline.

The vast majority of variants have recent origins in mammals, some carrying out functions unique to the germline (Fig. 2). Some of these newcomers arose multiple times from the same parental histone during evolution, as seen for H3mms in mouse, or H3.5 and H3.Y/X in primates. In such cases, it is tempting to speculate that recurring gene duplications helped resolve incompatible ancestral functions carried by a single parental histone, as previously observed in flies (Kursel and Malik 2017). Perhaps also as a result of conflicting selective forces during reproduction, many variants got lost along specific mammalian lineages — e.g., H2B.K and H2B.N in mouse, or H2A.Ls and H1.9 in primates — (Su et al. 2013; Molaro et al. 2018; Raman et al. 2022) (Fig. 2).

Finally, novel mammalian germline histones are particularly prone to positive selection. These include recently evolved sex-specific H2Bs, short H2As, H1T, and H1.9 (Ponte et al. 1998; Su et al. 2013; Molaro et al. 2018; Raman et al. 2022). While their evolutionary trajectories differ from cenH3, these signatures might also reveal ongoing germline genetic conflicts. In the case of the short H2A variant H2A.B, there is functional evidence supporting that parental antagonism or sexual conflict could drive its rapid evolution (Soboleva et al. 2017; Moretti et al. 2017; Molaro et al. 2018, 2020). It is interesting to note that these rapidly evolving histone families also display high gene turnover, perhaps further supporting the evolutionary arms races hypothesis (Fig. 2). However, the nature of these rapidly evolving functions and selective forces remains unknown for most variants.

In conclusion, with ongoing efforts to study histone-based parental-effects during reproduction, and the increasing interest in mapping their evolutionary histories, we can only predict that germline histones still have many spin-offs and sequels ready to hit the screen.

## Data Availability

Not applicable.
